# Methods for detecting *Gemmata* spp. bacteremia in the microbiology laboratory

**DOI:** 10.1186/s13104-017-3119-2

**Published:** 2018-01-08

**Authors:** Jacques-Robert Christen, Edwin Edmond, Michel Drancourt

**Affiliations:** 1 URMITE, UMR CNRS 7278, IRD 198, INSERM 1095, IHU Méditerranée Infection, Aix Marseille Université, 19-21 Bd Jean Moulin, 13005 Marseille, France; 2 Department of Infectious Diseases and Tropical Medicine, Laveran Military Teaching Hospital, Marseille, France

**Keywords:** *Gemmata*, Planctomycetes, Bacteremia, *Gemmata massiliana*, *Gemmata obscuriglobus*, PCR, Culture

## Abstract

**Objective:**

*Gemmata* bacteria are fastidious, Gram-negative and aerobic. The only representatives are *Gemmata obscuriglobus* and *Gemmata massiliana*. These Planctomycetes appear to be a part of human digestive tract microbiome, and *G. massiliana* has been isolated from water. Further specific detection in the blood of two patients with febrile neutropenia suggests that *Gemmata* bacteremia may remain under-documented. The objective of this study was to develop an effective protocol to document *Gemmata* spp. bacteremia in the laboratory. Using mock-infected and control blood specimens, three methods for detecting *Gemmata* bacteremia, namely, automated microbial detection, culture on solid medium, and quantitative polymerase chain reaction (PCR), have been developed and studied.

**Results:**

*Gemmata* spp. were undetected by automated blood culture system but culturing mock-infected blood on Caulobacter agar detected ≥ 10^2^ *G. obscuriglobus* bacteria/mL and ≥ 10^4^ *G. massiliana* bacteria/mL. Specific real-time PCR detected 10^2^ *Gemmata* bacteria/mL. These protocols may be used to investigate the epidemiology of *Gemmata* spp. bacteremia.

**Electronic supplementary material:**

The online version of this article (10.1186/s13104-017-3119-2) contains supplementary material, which is available to authorized users.

## Introduction

Bacteria of the genus *Gemmata* belong to the PVC superphylum (*Planctomycetes*–*Verrucomicrobia*–*Chlamydia*), phylum Planctomycetes [1]. These Gram-negative fastidious bacteria are resistant to most of the routinely used antibiotics [2]. Intracellular compartmentalization and multiplication by budding are two further remarkable characteristics of Planctomycetes and *Gemmata* [3].

The only two cultured *Gemmata* representatives are *Gemmata obscuriglobus* [1] and *Gemmata massiliana* [4]. Specific DNA sequences have been detected in the digestive microbiota [5]. The PVC member *Akkermansia muciniphila* has been directly sequenced from stools [6, 7] and cultured [8]. DNA sequences detected by polymerase chain reaction (PCR) amplification-sequencing in the blood of two patients with febrile neutropenia were more closely related to *Gemmata* spp. sequences [9]. However, detection of *Gemmata* bacteria in blood are currently limited to PCR-sequencing methods [9] which cannot be routinely implemented to investigate the epidemiology of *Gemmata* bacteremia [10]. *Gemmata* DNA is not detected by PCR targeting the 16S ribosomal ribonucleic acid (rRNA) routinely used in microbiology laboratory [11]. This so-called “universal” bacterial gene does have sequence variations among several bacteria genera, and the “universal” primers thought to target any bacterial 16S rRNA gene do not hybridize with the specific *Gemmata* 16S rRNA gene sequence.

The objective of this study was to develop tools to be further implemented in the routine clinical microbiology to detect *Gemmata* spp. bacteremia and further assess its epidemiology.

## Main text

### Mock-infected blood samples preparation

*G. obscuriglobus* (DSM 5831^T^) and *G. massiliana* (DSM 26013^T^) purchased from the Deutsche Sammlung von Mikrorganismen and Zellkuturen (Braunshwing, Germany) were plated on Caulobacter solid medium containing 2 g/L Bacto peptone (Sigma-Aldrich, Saint-Quentin Fallavier, France), with 1 g/L yeast extract (Sigma-Aldrich) and 0.2 g/L MgSO_4_ heptahydrate (Sigma-Aldrich) at 30 °C for 21 days [12]. Growing colonies were identified by matrix-assisted laser desorption ionisation-time of flight mass spectrometry (MALDI-TOF–MS) (Bruker Daltonics, Weissemburg, France) and a local database able to identify Planctomycetes thanks to a reproducible, unique protein profile comprising 23–39 peaks ranging in size from 2403 to 12,091 Da and differentiating the different species [13, 14] and specific PCR- sequencing of the Planctomycetes 16S rRNA [5, 15]. A 10^6^ bacteria/mL suspension was prepared in Hank’s balanced salt solution (HBSS) (Gibco, Waltham, MA) for each *Gemmata* species by using KOVA Glasstic Slides 10 (Hycor Biomedical, Indianapolis, IN) for counting viable bacteria after staining with Trypan blue (vital staining) (Eurobio, Courtaboeuf, France). Whole unqualified human blood was obtained from the Etablissement Français du Sang, Marseille, France. This procedure requires no specific ethical agreement in France as these anonymized blood specimens are regarded as left-over specimens.

The level of *Gemmata* spp. bacteremia is unknown, and we used a detection threshold of 10^4^ bacteria/mL of blood corresponding to reported blood inoculums for other Gram-negative bacteria such as *Klebsiella pneumoniae* or *Aeromonas hydrophila* [16]. A 10^4^ bacteria/mL blood specimen was prepared by pouring 5 mL blood and 50 µL the 10^6^ bacteria/mL HBSS suspension in an aerobic blood culture bottle (Versatrek, Waltham, USA); or by gently pouring 3 mL blood and 30 µL of the bacterial suspension into an ethylenediaminetetraacetic acid (EDTA) tube or a heparinized tube (BD Diagnostics, Le Pont de Claix, France). Heparin and EDTA are used as anticoagulant molecules preventing clot formation, thus allowing working with whole blood. All these manipulations were performed in quadruplicate using the prepared solutions of HBSS in order to obtain mock-infected blood containing 10^3^, 10^2^ and 10^1^ bacteria/mL of blood and negative controls incorporating the same volume of non-inoculated HBSS.

### Automated blood culture system

Mock-infected and negative control bottles were incubated in the Versatrek blood culture machine at 37 °C with constant stirring for a total of four 7-day runs. After a 7-day incubation period, 0.1 mL of the solution sampled in half of the bottles was plated on Caulobacter agar for subculture as described below in order to look for a bacterial persistence or growth that would not have been detected by the automated system. The same was done after 14 days of incubation for the remaining bottles.

### Culture on solid medium

EDTA and heparinized tubes were incubated immediately after preparation at 37 °C for 24–72 h to prevent misinterpretation by immediate plating after the inoculation of the tubes with bacteria and to look for bacterial growth in the tubes. Then, 0.1 mL of each tube was plated onto Caulobacter agar incubated at 30 °C for 21 days [1]. The number of colonies was determined weekly by visual inspection of plates.

### Development of a Gemmata specific qPCR system

A real-time PCR specific to this work was designed for detecting *Gemmata* spp. *rpo*B gene. We chose *rpo*B gene as one of the few universal bacterial genes whose sequence has been shown to accurately identify closely related bacterial species [17]. Primers and probes were selected using primer3 software [18, 19], primerBlast [20] and The Sequence Manipulation Suite [21]. Primers and probe specificity was tested in silico by a BLAST analysis against all sequences deposited in GenBank database (May, 2016). The specificity was then experimentally assessed using the DNA extracted from *G. massiliana*, *G. obscuriglobus* and 90 other bacterial species (Additional file 1). Three negative controls (containing the PCR mix without DNA) and two positive controls (containing the mix and *G. massiliana* DNA or *G. obscuriglobus* DNA) were used for the validation of the results. Real-time PCR assays were conducted in 20 µL-volume containing 5 µL DNA, 10 µL PCR mix (Eurogentec, Angers, France), 3.5 µL sterile water, 0.5 µL the forward primer 5′-GCAAGCTCAACTCGCTCAAC-3′ (40 nmol/L), 0.5 µL the reverse primer 5′-CTTCGAGATGACGCCCTTGT-3 (40 nmol/L), (Eurogentec) and 0.5 µL TaqMan MGB probe labeled with the fluorophore FAM 6 5′-ATGGTGAAGGTCTACGTCGC-3′ (6 nmol/L) (Applied Biosystems, Courtaboeuf, France). The amplification cycle consisted of 2-min pre-incubation at 50 °C followed by 15-min denaturation at 95 °C and 46 cycles of 30 s at 95 °C and 1 min at 58 °C. The sample was cooled at 45 °C for 30 s. The qPCR was used to quantify the detection of *Gemmata* organisms in mock-infected blood specimens and negative controls. All DNA extractions were performed on EZ1 advanced XL using EZ1 DNA Investigator Kit (Qiagen, Courtaboeuf, France), suitable for purification of DNA from whole blood with heparin or EDTA.

### Statistical methods

The statistical analyses were done using R Statistical Software (Foundation for Statistical Computing, Vienna, Austria). We used the Fisher’s exact test. For all statistical analyses, a p < 0.05 was considered significant.

### Results

#### Automated blood culture system

No bacterial growth was detected by the automated blood culture system, regardless of the *Gemmata* species, the bacterial concentration (10^5^, 10^4^, 10^3^, 10^2^, or 10^1^ bacteria/mL of blood) and the incubation time (7, 14 or 30 days). No growth was detected for negative controls incubated during 7, 14, or 30 days.

#### Culture on Caulobacter medium

No growth was detected in the negative control plates and in the plates inoculated with the blood culture bottles incubated for 7 or 14 days. Likewise, no *Gemmata* colony was observed in the plates inoculated with blood from EDTA or heparin tubes incubated for 72 h at 37 °C before plating. *G. massiliana* colonies were observed on 5/32 agar plates seeded with mock-infected EDTA or heparin blood tubes inoculated with 10^4^ bacteria/mL incubated for 24 h, but not for the other tested inocula. Significantly more *G. obscuriglobus* (18/32, detection up to an initial concentration of 10^2^ bacteria/mL) were detected (p = 0.004, 95% confidence interval (IC 95%) [1.53–21.52]). Growth was observed after a 14-day incubation period for *G. obscuriglobus* and a 21-day incubation period for *G. massiliana* (Table 1).

**Table 1:** Number of colonies observed after a 24-h inoculation at 37 °C on Caulobacter medium of *Gemmata* sp. mock-infected blood in EDTA and heparin tubes

	*Gemmata massiliana* EDTA tubes			*Gemmata massiliana* Heparin tubes			*Gemmata obscuriglobus* EDTA tubes			*Gemmata obscuriglobus* Heparin tubes		
Initial concentration (bacteria/mL)	10^2^	10^3^	10^4^	10^2^	10^3^	10^4^	10^2^	10^3^	10^4^	10^2^	10^3^	10^4^
Culture results (CFU/mL)	0	0	200	0	0	320	0	90	300	70	270	420
	0	0	0	0	0	0	0	170	450	70	180	570
	0	0	480	0	0	0	†	0	400	20	410	550
	0	0	250	0	0	980	30	†	380	0	50	500

No colony was observed for an initial concentration of 10^1^ bacteria/mL

CFU, colony-forming unit; †, contaminated

#### qPCR

When we tested the specificity of the qPCR here reported, *G. massiliana* (cycle threshold: 26.6) and *G. obscuriglobus* (cycle threshold: 27.1), DNA was detected, and the qPCR was negative for all the 90 other DNA-tested bacterial species (Additional file 1). No *Gemmata* spp. DNA was detected from any of the negative controls while positive controls were detected in all experiments with Ct values of 27.97 ± 0.76.

Nine out of sixteen qPCR performed on samples extracted from blood culture bottles were positive after a 7-day incubation period for *G. massiliana* and 5/16 for *G. obscuriglobus* (p = 0.285, IC 95% [0.54–15.45]). After a 14-day incubation period, only 6/32 qPCR reactions were positive (p = 0.058, IC 95% [0.97–12.62]) (Table 2).

**Table 2:** Ct values observed after qPCR amplification of *Gemmata* sp. DNA extracted from mock-infected blood culture bottles

Initial concentration (bacteria/mL)	*Gemmata massiliana* Incubation: 7 days				*Gemmata obscuriglobus* Incubation: 7 days				*Gemmata massiliana* Incubation: 14 days				*Gemmata obscuriglobus* Incubation: 14 days			
	10^1^	10^2^	10^3^	10^4^	10^1^	10^2^	10^3^	10^4^	10^1^	10^2^	10^3^	10^4^	10^1^	10^2^	10^3^	10^4^
qPCR results (CT)	38.1	35.7	–	36.1	–	–	35.6	–	–		–	33.7	–	–	–	–
	–	–	–	35.0	–	–	36.9	38.3	–	–	–	35.1	–	–	–	38.3
	–	35.5	36.2	33.2	–	–	–	–	–	–	–	36.0	–	–	–	37.4
	–	–	34.1	33.6	–	38.1	37.0	–	–	–	–	32.1	–	–	–	–

–, no amplification detected; CT, cycle threshold

**Fig. 1:**
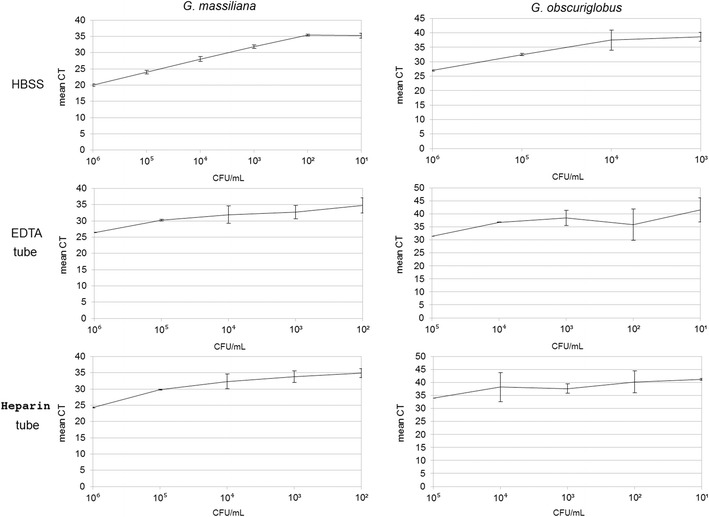
qPCR-calibration curves of *G. massiliana* and *G. obscuriglobus* in blood. The nature of the blood sample and negative control is indicated in the left margin. *x* axis, value of the inoculums in CFU/mL; *y* axis, Ct value (mean of 3 experiments)

Twenty-nine out of 32 qPCR performed on samples extracted from EDTA and heparin tubes were positive for *G. obscuriglobus* whereas 23/32 were positive for *G. massiliana* (p = 0.107, IC 95% [0.80–23.72]). There was no detection of *G. massiliana* in the tubes containing 10^1^ bacteria/mL of blood. For the other inocula, 23/24 detections were positive in qPCR (Additional file 2: Table S1). qPCR results allowed to draw calibration curves for the two *Gemmata* species (Fig. 1).

Overall, qPCR detected significantly more (52/64) *Gemmata* spp.-mock-infected blood in EDTA and heparin tubes than in culture on Caulobacter medium (23/64, p < 0.001, IC 95% [3.22–18.98]).

## Discussion

We previously reported the PCR-sequencing-based detection of *Gemmata* sequences in whole blood collected from two patients with undocumented febrile neutropenia [6]. This unique observation led us to the hypothesis according to which *Gemmata* bacteria could be responsible for opportunistic bacteremia after digestive translocation in neutropenic patients [5]. To further explore this hypothesis, it was necessary to develop additional laboratory tools which could be used for the routine screening of blood culture bottles, in order to further assess the epidemiology of *Gemmata* bacteremia in these patients.

Here, we confirm that *Gemmata* bacteria are not detectable in whole blood by automated microbial detection systems for blood cultures. *Gemmata* bacteria incubated in these blood bottles for 7 days did not grow, since no bacterial growth was observed on appropriate Caulobacter agar medium. It is possible that the incubation temperature (37 °C) in the automated system may not have been uniformly optimal throughout, but it does not explain the absence of growth in *Gemmata* spp which are able to grow at 30 °C [22]. The continuous agitation of the blood bottles may be an additional deleterious factor, but that does not explain the lack of *Gemmata* growth in non-agitated EDTA and heparin tubes incubated for 72 h at 37 °C. The most likely hypothesis is that a 7-day contact of *Gemmata* bacteria with whole blood collected from non-aplastic blood donors allowed for blood components to elicit bactericidal activity against *Gemmata* bacteria, at 37 °C under agitation. Nevertheless, our observations coupled with the lack of any reported case of culture-proven *Gemmata* bacteremia, even in aplasic patients, strongly suggest that blood culture automatons may not be able to accurately detect *Gemmata* in blood.

Therefore, we aimed at designing further culture-based detection of *Gemmata* bacteremia and showed that direct plating of freshly collected blood on Caulobacter agar medium could allow to detect 10^4^ bacteria/mL of blood. However, the 21-day delay needed for *Gemmata* growth limits this method as a first-line screening of blood cultures.

We therefore developed a *Gemmata*-specific qPCR as a screening method. After confirming the specificity of this method for the detection of *Gemmata* DNA, we observed that this technique offered the highest sensitivity in our study, allowing for the detection of 10^2^–10^4^ *Gemmata* bacteria/ml of blood drawn in either EDTA or heparinized tubes.

### Conclusions

This methodological study sets up the bases for the routine detection of *Gemmata* bacteremia and we are now routinely screening aerobic blood culture bottles collected in aplastic patients by using the *rpo*B gene sequence-based qPCR here reported; followed by plating qPCR-positive blood specimens on Caulobacter agar medium as reported here. This technical report may prompt the search for blood-borne *Gemmata* species in other clinical microbiology laboratories serving clinical departments for aplastic patients.

## Limitations

The results here exposed come from an experimental study and thus depend on the experimental conditions here reported, including the culture media and the inocula. Therefore, the generalization of observations here made with one of the few commercially available blood-culture automatons compared to the other routinely available systems (Bactec, Becton–Dickinson, and BacT-ALERT, bioMérieux, Marcy-l’Etoile, France) deserves to be verified by other studies. These results are a basis to investigate clinical specimens, but the experimental conditions here reported may differ from the actual conditions of clinical specimens.

## Additional files

**Additional file 1.** 93 bacterial species from which DNA was extracted to assess the specificity of qPCR in vitro.

**Additional file 2: Table S1.** qPCR performed on samples extracted from EDTA and heparin tubes.

## Abbreviations

DNA: deoxyribonucleic acid
RNA: ribonucleic acid
rRNA: ribosomal ribonucleic acid
CSUR: Collection de souches de l’Unité des Rickettsies (Marseilles, France)
CT: cycle threshold
DSMZ: Deutsche Sammlung von Mikrorganismen and Zellkuturen (Braunshwing, Germany)
HBSS: Hank’s balanced salt solution
MALDI-TOF: Matrix Assay Laser Desorption Ionisation-Time Of Flight
PCR: polymerase chain reaction
qPCR: real-time PCR
spp.: species pluralis
CFU: colony-forming unit
USA: United-States of America
