# Supplementary material for: Methods for detecting Gemmata spp. bacteremia in the microbiology laboratory
Source: BMC Res Notes. 2018 Jan 8;11:11. doi: 10.1186/s13104-017-3119-2 (PMC5759251; doi:10.1186/s13104-017-3119-2)
Supplement: Supplementary file 1 — Additional file 1. 93 bacterial species from which DNA was extracted to assess the specificity of qPCR in vitro. [file 13104_2017_3119_MOESM1_ESM.docx]

**Additional material: 93 bacterial species from which DNA was extracted to assess the specificity of qPCR *in vitro*.**

*Achromobacter* spp*.*

*Acinetobacter baumanii*

*Acinetobacter baylyi*

*Acinetobacter calcoaceticus*

*Acinetobacter haemolyticus*

*Acinetobacter johnsonii*

*Acinetobacter junii*

*Acinetobacter lwoffii*

*Acinetobacter parvus*

*Acinetobacter radioresistens*

*Arcanobacterium haemolyticum*

*Bacillus brevis*

*Bacillus cereus*

*Bacillus megaterium*

*Bacillus sphaericus*

*Bacteroides fragilis*

*Bartonella henselae*

*Bartonella quintana*

*Bordetella bronchoseptica*

*Bordetella holmesii*

*Burkholderia cenocepacia*

*Burkholderia cepacia*

*Capnocytophaga*

*Corynebacterium diphtheriae*

*Corynebacterium tuberculostearicum*

*Enterobacter aerogenes*

*Enterobacter cloacae*

*Enteroccocus faecium*

*Enterococcus faecalis*

*Escherichia coli*

*Finegoldia magna*

*Fusobacterium necrophorum*

*Fusobacterium nucleatum*

*Haemophilus agni*

*Haemophilus aphrophilus*

*Haemophilus influenzae*

*Haemophilus parahaemolyticus*

*Haemophilus parainfluenzae*

*Haemophilus parasuis*

*Klebsiella pneumoniae*

*Lactobacillus* spp*.*

*Legionella pneumophila*

*Legionella rubriculencens*

*Legionella taurinensis*

*Leptospira alexanderi/ranarum pingek vang*

*Leptospira genomospecies3/wazholland*

*Leptospira genomospecies5/saopolo*

*Leptospira inadai/lyme*

*Leptospira kirschneri/cynopteni3522C*

*Leptospira meyeri/ranarum*

*Leptospira noguchi/panama*

*Leptospira weilli/celladoni*

*Leptospira wolbachi/codice*

*Listeria monocytogenes*

*Moraxella catarrhalis*

*Moraxella catarrhalis*

*Mycoplasma genitalium*

*Mycoplasma hominis*

*Neisseria gonorrhoeae*

*Neisseria meningitidis*

*Nocardia farcinica*

*Parabacteroides*

*Prevotella spp.*

*Prevotella denticola*

*Prevotella melaninogenica*

*Propionibacterium acnes*

*Pseudomonas aeruginosa*

*Rothia* spp*.*

*Salmonella enterica subsp.salamae*

*Serratia marcescens*

*Staphylococcus aureus*

*Staphylococcus epidermidis*

*Staphylococcus hominis*

*Stenotrophomonas maltophilia*

*Streptococcus agalactiae*

*Streptococcus anginosus*

*Streptococcus constellatus*

*Streptococcus gallolyticus*

*Streptococcus gordonii*

*Streptococcus intermedius*

*Streptococcus mitis*

*Streptococcus mitis*

*Streptococcus mutans*

*Streptococcus oralis*

*Streptococcus pneumoniae*

*Streptococcus pseudopneumoniae*

*Streptococcus pyogenes*

*Streptococcus salivarius*

*Streptococcus sanguinis*

*Veillonella* spp*.*
