# Supplementary material for: Methods for detecting Gemmata spp. bacteremia in the microbiology laboratory
Source: BMC Res Notes. 2018 Jan 8;11:11. doi: 10.1186/s13104-017-3119-2 (PMC5759251; doi:10.1186/s13104-017-3119-2)
Supplement: Supplementary file 2 — Additional file 2: Table S1. qPCR performed on samples extracted from EDTA and heparin tubes. [file 13104_2017_3119_MOESM2_ESM.docx]

**Additional Table S1**: qPCR performed on samples extracted from EDTA and heparin tubes.

|  | *Gemmata massiliana* EDTA tubes | | | | *Gemmata massiliana* Heparin tubes | | | | *Gemmata obscuriglobus* EDTA tubes | | | | *Gemmata obscuriglobus* Heparin tubes | | | |
| --- | --- | --- | --- | --- | --- | --- | --- | --- | --- | --- | --- | --- | --- | --- | --- | --- |
| Initial concentration (bacteria/mL) | 10^1^ | 10^2^ | 10^3^ | 10^4^ | 10^1^ | 10^2^ | 10^3^ | 10^4^ | 10^1^ | 10^2^ | 10^3^ | 10^4^ | 10^1^ | 10^2^ | 10^3^ | 10^4^ |
| qPCR results (CT) | - | 32.0 | 33.2 | 30.8 | - | 35.6 | 32.2 | 26.9 | - | 41.5 | 32.1 | 36.6 | 41.4 | 40.3 | 38.6 | 36.9 |
|  | - | 35.8 | 31 | 28.8 | - | 36.3 | 33.0 | 33.9 | 42.0 | 40.3 | 32.0 | 38.1 | 41.2 | 40.6 | 38.4 | 35.7 |
|  | - | 36.4 | 31.8 | 29.3 | - | 33.3 | 35.3 | 29.3 | 41.0 | 35.4 | 30.8 | 34.0 | - | 39.8 | 30.7 | 39.5 |
|  | - | - | 31.3 | 34.2 | - | 34.5 | 32.4 | 30.6 | - | 37.0 | 30.3 | 34.1 | 41.2 | 40.2 | 38.6 | 34.1 |

-: no amplification detected CT: cycle threshold
